# *Toxoplasma gondii *infection in slaughter pigs in Serbia: seroprevalence and demonstration of parasites in blood

**DOI:** 10.1186/1297-9716-42-17

**Published:** 2011-02-01

**Authors:** Ivana Klun, Marija Vujanić, Hélène Yera, Aleksandra Nikolić, Vladimir Ivović, Branko Bobić, Siniša Bradonjić, Jean Dupouy-Camet, Olgica Djurković-­Djaković

**Affiliations:** 1 National Reference Laboratory for Toxoplasmosis, Centre for Parasitic Zoonoses, University of Belgrade Institute for Medical Research, Dr. Subotića 4, 11129 Belgrade, Serbia; 2 Laboratory of Parasitology-Mycology, Hôpital Cochin, AP-HP, Université Paris Descartes, 27 rue du Faubourg St Jacques, 75014 Paris, France; 3 Veterinary Directorate, Ministry of Agriculture, Forestry and Water Management of Serbia, Omladinskih brigada 1, 11000 Belgrade, Serbia

## Abstract

A seroepizootiological study of *Toxoplasma gondii *infection involving a total of 488 slaughter pigs (468 market-weight pigs and 20 sows) in the Belgrade area, also included examination of the presence of *T. gondii *in the blood. Blood sampled at the slaughter line was examined for specific antibodies by modified direct agglutination, and blood clots of those seropositive at titres of 1:50-1:12800 were bioassayed in mice. The overall seroprevalence was 9.2%, significantly higher (*p *= 0.0063) in sows (30.0%) than in market-weight pigs (8.3%). Amongst the 22 bioassays performed, a total of 16 (72.7%) were positive, by observation of *T. gondii *cysts (12), seropositivity (7, including 3 in which cysts were not detected), and/or detection of *T. gondii *DNA by real-time PCR (12, including one otherwise negative). The positive bioassays originated from the blood of 12 market-weight pigs and 4 sows. Despite a general increase in the rate of demonstration of *T. gondii *with the increase in the specific antibody level, the association was not significant (*p *= 0.101). The risk of infection was 41-fold increased in sows vs market-weight pigs, and 15-fold in pigs from smallholders' finishing type farms vs those from large farrow-to-finish farms. The presence of viable *T. gondii *in a proportion of the samples indicates that some of the pigs had an active parasitaemia at the time of slaughter, which, along with the seroprevalence established, points to a potential source of human infection in Serbia. This is the first report on parasitaemia in naturally infected swine.

## Introduction

Toxoplasmosis is a globally distributed zoonosis with a clinical impact in the unborn fetus and in the immunosuppressed individual.

Consumption of undercooked meat/meat products has been well established as a major risk factor for human *Toxoplasma gondii *infection worldwide [1], including in Serbia [2,3]. In a recent study conducted to establish the risk of human *T. gondii *infection from food animals in Serbia, we found a seroprevalence of 76.3% in cattle, 84.5% in sheep and 28.9% in pigs [4]. Although the prevalence in swine was comparatively lower, pork is generally a major meat source of human infection [1], and is by far the meat type mostly consumed in the country, accounting for (including pork products) nearly 50% of all meat consumed [5].

Whereas ingestion of the *T. gondii *bradyzoite (in tissue cysts) form is a major route of infection, bradyzoites only develop following a brief stage characterized by tachyzoites in the bloodstream (parasitaemia). However, no data are available on parasitaemia in naturally infected pigs. Therefore, within a seroepizootiological study of slaughter pigs in the Belgrade area conducted to further analyze the potential risk of pork for human infection, we took advantage of the availability of full blood samples to also examine the presence of *T. gondii *in swine blood.

## Materials and methods

### Study population and collection of samples

The study involved a total of 488 pigs (468 market-weight pigs and 20 sows), sampled in the three main Belgrade abattoirs between March and May 2007. These abattoirs are located in the wider city area (up to 20 km from downtown), and process, among them, around 1000 pigs per day. The animals were sampled at the slaughter line, during thoracic stick exsanguination, by members of the research team who collected blood samples (8 mL each into a sterile tube) at a rate of 20-50 samples per visit to the abattoir (15 total visits).

Samples were transported on ice to the IMR laboratory the same day. After centrifugation (2000 rpm for 20 min), the sera were immediately tested for *T. gondii *antibodies. Blood clots were stored at 4°C until the reading of the serological test the following day, the result of which determined whether a clot was to be further processed.

### Collection of epizootiological data

The pigs processed at the sampled abattoirs originated from northern Serbia and the Belgrade District, and from both large farrow-to-finish and smallholders' finishing type farms. Data collected at the abattoirs were obtained from the health certificates and included age group and farm type. Pigs were classified by age as market-weight age (< 8 months) or adults/sows (≥ 8 months). All sows were from farrow-to-finish farms, where we collected data on their parity (as an indication of age). The sows were excluded from reproduction at 2-4 weeks post partum, and their mean parity was 4.5 ± 2.8 (range 1-10). They all had group access to outside pens with dirt during the "weaning to service" period and during pregnancy for up to one week before farrowing, when they were placed in individual inside farrowing stalls.

### Study design

Blood samples collected at the slaughter line were immediately examined for *T. gondii *antibodies, and clots of those seropositive were bioassayed in mice. These mice were also tested serologically, and their brain tissue examined for morphologically recognizable *T. gondii *cysts and presence of *T. gondii *DNA.

Based on the reasoning that animals with higher specific antibody levels have a greater chance of being associated with acute infection, it was originally planned to attempt isolation of *T. gondii *from all pigs seropositive at a titre ≥ 1:400. However, the initial results revealed that these were few, and thus, the protocol was adapted to bioassay animals positive at titres of ≥ 1:50.

The study protocol was approved by a local (Institute for Medical Research) Ethics Committee.

### Serology

*T. gondii-*specific IgG antibodies (in both pig and mouse sera) were detected by the modified agglutination test (MAT) as described by Desmonts and Remington [6]. Formalin-fixed whole RH tachyzoites used as antigen were obtained from Dr Philippe Thulliez (IPP, Paris, France). Sera were serially two-fold diluted starting at 1:25. Positive and negative controls (a laboratory standard prepared according to the WHO reference serum assessed to contain 1000 IU/mL and PBS buffer, respectively) were included in each run. Sera reactive at ≥ 1:25 were considered positive.

### Bioassay

For parasite isolation, female Swiss-Webster mice (Medical Military Academy Animal Research Facility, Belgrade, Serbia) were used. Mice, weighing 18-20 g at the beginning of each experiment, were housed at the IMR Animal Research Facility and offered regular mouse feed and drinking water *ad libitum*. Naïve mice are occasionally examined for *T. gondii *at random, and none have ever been shown to be infected.

Bioassays were performed following the routine procedure in our laboratory [7]. Blood clots were homogenized with residual serum with forced passes through an 18-gauge needle, and 500 μL suspensions, together with 100 μL gentamicin solution (0.8 mg/mL), were inoculated intraperitoneally into four naïve mice each.

All mice were monitored daily over a period of six weeks; the peritoneal fluid of those succumbing was examined for the presence of *T. gondii *tachyzoites, and microbiological culture of peritoneal fluid was performed. After six weeks, mice were euthanized and blood samples were taken for serology, whereas the brains were removed for cyst enumeration and DNA extraction.

Brains (mean volume 400 μL) were homogenized with 1 mL of saline each with forced passes through an 18-gauge needle. For cyst enumeration, 25 μL of the brain suspensions were placed on slides and counted under a phase-contrast microscope (by three experienced researchers). The number of cysts per brain was calculated by multiplying the number counted in four drops by 14, giving a threshold sensitivity of our method of 14 cysts per brain. A bioassay was considered positive for cysts if at least one *T. gondii *cyst was detected in any of the four inoculated mice.

### Real-time PCR

DNA extraction and amplification of the *T. gondii *529-bp element (GenBank accession number AF146527) were performed according to the protocol described by Talabani et al. [8], adapted to our substrate. Extraction of DNA from 200 μL-samples of homogenized murine brain tissue was carried out using QIAamp DNA Mini Kit (Qiagen, Hilden, Germany), according to the manufacturer's tissue protocol. The amplification and detection were performed on a Mastercycler ep realplex^4 ^(Eppendorf AG, Hamburg, Germany), using TaqMan Universal Master Mix with uracil N-glycosylase (Applied Biosystems, Foster City, CA, USA). DNA extracts were tested three times by real-time PCR, undiluted with and without internal control, and diluted 1:10 with internal control (instead of 1:2 as described by Talabani et al. [8]). Each PCR run included a negative extraction control (sterile distilled water), a positive extraction control diluted to obtain 0.5 and 5 *T. gondii *genome equivalents per reaction, a negative PCR control (a DNA extract previously found to be negative), and a positive PCR control (a DNA extract previously found to be positive). A sample was declared positive if at least one of the triplicates was positive.

### Statistical analysis

The association of the specific antibody levels (continuous variable) in swine with the demonstration of *T. gondii *in blood was analyzed by logistic regression. The modalities of demonstration included "cyst-positive bioassay", "either cyst- or MAT-positive bioassay" (only MAT-positive was not taken into account due to a small sample and for lack of relevance), "qPCR-positive bioassay", or "summary demonstration" (denoting the total number of positive findings by any technique). The overall fit of the logistic regression model was assessed using the Hosmer-Lemeshow goodness-of-fit statistics.

The difference in the seroprevalence between the age categories was analyzed by Fisher's exact test. This test, or, where appropriate, the chi-square test, was also used to analyse the influence of examined factors as independent categorical variables on *T. gondii *seroprevalence. Variables significant at *p *≤ 0.1 at the 95% confidence level were tested for collinearity and were selected for inclusion in the multivariate logistic regression model. The overall fit of the model was again assessed using the Hosmer-Lemeshow goodness-of-fit statistics. The results are presented as adjusted odds ratios (OR) with 95% confidence intervals (95% CI). The differences in parity between seropositive and seronegative sows were examined by the Student's *t*-test.

The level of significance was 5%. All statistics were performed using the SPSS version 11.5 statistical package (SPSS Inc., Chicago, IL, USA).

## Results

The overall seroprevalence in the series of 488 examined pigs was 9.2%, with specific antibody levels ranging from 1:25 to 1:12800. The prevalence was significantly higher (*p *= 0.0063, Fisher's exact test) in sows (30.0%) than in market-weight pigs (8.3%).

Bioassays were performed with clotted blood from 22 of the 45 seropositive pigs (~ 50%, which was a number both statistically sound and capacity-wise feasible) of those with specific antibody levels ranging from 1:50 to 1:12800. The results of the bioassays are presented in Table 1. *T. gondii *cysts, ranging in diameter from 17 to 58 μm, were observed in 18 mice from 12 (54.5%) bioassays, at low numbers (range 14-56 per brain). Four cyst-positive bioassays tested positive in MAT, with only one mouse seropositive in each. In contrast, serology was positive in three bioassays in which cysts were not observed; notably, two of these originated from pigs with the highest specific antibody levels detected (1:12800 and 1:3200).

**Table 1:** Outcome of bioassays of blood from 22 seropositive pigs sampled at Belgrade abattoirs

Number	MAT titre (pig sera)	Mice with cysts/mice examined (n)	Seropositive mice/mice examined (n) (MAT titre)	Real-time PCR (mouse brain)
Market-weight				
1	1:1600	0/1^a^	0/1^a^	ND
2	1:200	1/4	0/4	+
3	1:400	2/3^a^	0/3^a^	+
4	1:400	3/4	0/4	+
5	1:12800	0/4	1/4 (1:100)	-
6	1:800	1/4	0/4	-
7	1:1600	0/3^a^	0/3^a^	+
8	1:3200	0/4	1/4 (1:50)	-
9	1:400	0/4	1/4 (1:25)	+
10	1:800	1/4	1^b^/4 (1:25)	+
11	1:50	1/4	1/4 (1:50)	+
12	1:400	1/4	0/4	+
13	1:50	0/4	0/4	-
14	1:50	0/4	0/4	-
15	1:200	0/4	0/4	-
16	1:400	1/4	0/4	+
17	1:200	0/4	0/4	-
Sows				
18	1:400	0/4	0/4	ND
19	1:100	1/4	0/4	+
20	1:1600	2/4	0/4	+
21	1:200	3/4	1^b^/4 (1:25)	+
22	1:800	1/4	1^b^/4 (1:25)	-

^a ^mice (a total of 5) died before time of sacrifice (*Erysipelothrix rhusiopathiae *isolated from peritoneal exudates of succumbed mice from bioassay no. 1)

^b ^seropositive mouse had *T. gondii *tissue cysts; ND, assay not done.

Real-time PCR for *T. gondii *DNA was performed in the mouse brain of 20 of the 22 bioassays (two were missed due to technical reasons). *T. gondii *DNA was detected in 12 (60%), 11 of which were positive, and in one otherwise negative bioassay. In summary, of the 22 bioassays performed, as many as 16 (72.7%) were positive by at least one technique (Table 2).

**Table 2:** Distribution of performed and positive bioassays according to specific antibody level in pigs

Number	Antibody titre (reciprocal)										Total
		
	25	50	100	200	400	800	1600	3200	6400	12800	
Pigs examined	9	11	1	6	7	4	3	2	0	2	45
Bioassays performed	-	3	1	4	6	3	3	1	-	1	22
Parasite demonstration	-	1	1	2	5^a^	3	2^b^	1^c^	-	1^c^	16

^a ^one bioassay sero- and qPCR-positive (no cysts found)

^b ^one only qPCR-positive

^c ^only seropositive

The rate of presence of *T. gondii *showed a general increase with the increase in the specific antibody level (33% at a titre of 1:50 vs 78.9% at titres of ≥ 1:100, and 50% at ≤ 1:200 vs 85.7% at ≥ 1:400) (Table 2). However, the analysis of the success of demonstration by modality ("summary demonstration" having the best model fit) according to the specific antibody level detected in the pig showed no association between parasite demonstration and the specific antibody level detected in the originating swine (*p *= 0.101, logistic regression).

The results were further analyzed according to the swine age and originating farm type. Twelve of the 16 positive bioassays originated from market-weight pigs (12/17, 70.6%) and 4 (4/5, 80%) from sows. All sows were from farrow-to-finish farms, but all market-weight pigs in which *T. gondii *was isolated were from smallholder finishing farms.

The analysis of the risk factors was performed in the whole group of 488 pigs. Factors associated with seropositivity in the univariate analysis were age group, farm type, and sampling site (abattoir) (Table 3). High collinearity was detected between "farm type" and "abattoir" variables (Cramer's V = 0.929), and since "abattoir" as a sampling site is not a biologically plausible factor of *T. gondii *infection in swine, age group and farm type were thus included in the multivariate logistic model. Analysis showed that age group and farm type were independently associated with *T. gondii *infection, with an almost 41-fold higher likelihood of infection in sows (*p *< 0.001) compared to market-weight hogs and gilts, and almost 15-fold higher in pigs of all ages (market-weight and sows) from smallholders' finishing type farms (*p *< 0.001) compared with those from farrow-to-finish farms (Table 3). The influence of age on the prevalence of *T. gondii *infection was also shown by a significantly higher (*p *= 0.032) parity of seropositive than of seronegative sows.

**Table 3:** *T. gondii*-specific antibodies in pigs according to age group, farm type and sampling site (abattoir), and results of univariate and multivariate logistic regression analysis

Factor	n	Prevalence (%)	95% CI	Univariate analysis			Multivariate analysis		
					
				OR	95% CI	*p*-value	Adjusted OR	95% CI	*p*-value
Age group									
Market-weight (< 8 months)	468	8.3	5.8-10.8	1.00		0.006	1.00		
Adult/sows (≥ 8 months)	20	30.0	9.9-50.1	4.71	1.72-12.96		40.71	7.51-220.55	< 0.001
Farm type									
Farrow-to-finish	212	3.8	1.2-6.4	1.00		< 0.001	1.00		
Smallholders' finishing	276	13.4	9.4-17.4	3.95	1.80-8.67		14.71	3.50-61.78	< 0.001
Abattoir*									
1	192	1.0	0.0-2.4	1.00		< 0.001			
2	182	15.9	10.6-21.2	18.00	4.23-76.64				
3	114	12.3	6.3-18.3	13.30	2.96-59.66				
Total	488	9.2	6.6-11.8						

* Not included in multivariate analysis

## Discussion

The results of this study showed a seroprevalence of 9.2% of *T. gondii *infection in a total of 488 swine from abattoirs in the vicinity of Belgrade. Antibody levels in the 45 seropositive animals ranged from 1:25 to 1:12800. Bioassays in mice performed with blood samples from 22 pigs seropositive at ≥ 1:50 showed biological and/or molecular evidence of *T. gondii *in as many as 16 (73%). Although there was a general increase in the rate of demonstration of *T. gondii *in the blood with the increase in the specific antibody level detected in swine, the association was not significant. This indicates that the presence of the organism in the blood may not be predicted on the basis of the specific antibody level detected in swine.

The overall prevalence of 9.2% in pigs from Belgrade abattoirs was three-fold lower than the 28.9% found in our recent study in a representative sample of 605 swine from throughout Serbia [4]. This difference may largely be attributed to the difference in the samples studied since the present one consisted of a large majority (96%) of market-weight pigs, who generally have a much lower prevalence than adult pigs.

Most importantly, viable parasites were demonstrated in a high proportion (73%) of the performed bioassays (among animals seropositive at ≥ 1:50), indicating that seropositive animals quite frequently had an active parasitaemia. This result may even be an underestimate, since isolation procedures were only performed in animals seropositive at ≥ 1:50, and *T. gondii *has been isolated from the tissues of pigs with specific antibody levels of 1:40 and 1:20 [9] and 1:16 [10], and even from seronegative ones [11,12].

In this study, it was not possible to correlate the findings in the blood with those in the tissues (which were not available due to economic and technological restrictions by the abattoirs). Previous studies on the demonstration of *T. gondii *in naturally infected pigs were performed in tissues including the heart, tongue, diaphragm and brain (but not blood), in which isolation of *T. gondii *ranged from 0.4% in Austria [13] and 1.1% in the Czech Republic [14] to 12.8% in Argentina [10] and 17% in the USA [15]. A study from Brazil showed isolation from the brain in even 50% but of only 12 examined animals [16]. When only tissues from seropositive animals were bioassayed, *T. gondii *was isolated in 25% in Brazil [17], 36.8% in the USA [12] and 40.5% in Portugal [9]. Viable *T. gondii *was also isolated from 0.3% retail pork samples by cat bioassay [18], and in 8.7% of examined retail sausages by mouse bioassay (based, however, largely only on the seropositivity of mice) [19].

The results presented here are the first to date demonstrating viable *T. gondii *from the blood of naturally infected pigs. Parasitaemia is considered to last no longer than a few weeks, but data are available that challenge this assumption. Early work had shown spontaneous recurrent parasitaemia in mice, rabbits and guinea-pigs up to eight months after experimental infection [20]. Parasitaemia lasting as long as 62 and 84 days has been reported after experimental infection in cattle and buffaloes [21,22]. Similarly, in goats, parasitaemia has been detected up to 64 days post experimental infection, without apparent relationship with specific antibody as detected by ELISA and Western blot [23]. In sheep, *T. gondii *DNA has been demonstrated 14 days after experimental infection, but has not been sought for later [24]. However, no data on the occurrence or duration of parasitaemia are available in naturally nor experimentally infected pigs. Thus, our observation of frequent parasitaemia in seropositive pigs warrants experimental investigation.

Detection of viable *T. gondii *in swine blood in this study, in the absence of technicalities such as cross-contamination (excluded by the facts that the cutting knife was always rinsed in water between uses on the following animals and that isolations did not come from consecutively slaughtered animals) or collection of parasites from severed tissues along the incision route (highly improbable given the short time the blood was in contact with the damaged tissues and the low likelihood of *T. gondii *cysts being located precisely along the particular random route) implies acute infection, or alternatively, reactivation or reinfection.

The rate of parasitaemia detected in pigs as well as the seroprevalence in Serbia reflect the level of contamination of the environment already established in our previous work [4]. This is now emphasized by a much higher frequency of infection in pigs from smallholders' than in large farrow-to-finish farms, and in sows than in market-weight animals. Importantly, all market-weight pigs in which *T. gondii *was isolated were from smallholders' finishing farms. Continuous exposure to *T. gondii *is also reflected by the older age of seropositive sows. Since tachyzoites naturally convert into bradyzoites within tissue cysts, the rate of parasitaemia, along with the seroprevalence established in slaughter pigs in Serbia, collectively point to a potential source of human infection, calling for action oriented at the production chain.

## Competing interests

The authors declare that they have no competing interests.

## Authors' contributions

IK and MV collected the samples and epizootiological data, and carried out the serological and molecular assays. HY and VI defined the protocol for the molecular assays and participated in the result analysis. IK, AN and BB carried out the bioassays. SB participated in the design and the organization of the field studies and in the statistical analysis. IK and ODjDj drafted the manuscript. JDC participated in the design of the study and in drafting the final version of the manuscript. ODjDj conceived the study, and participated in its design and coordination. All authors read and approved the final manuscript.
